# Self-Rated Physical Health and Unmet Healthcare Needs among Swedish Patients in Opioid Substitution Treatment

**DOI:** 10.1155/2019/7942145

**Published:** 2019-04-15

**Authors:** Katja Troberg, Anders Håkansson, Disa Dahlman

**Affiliations:** ^1^Lund University, Faculty of Medicine, Department of Clinical Sciences Lund, Psychiatry, Lund, Sweden; ^2^Malmö Addiction Centre, Region Skåne, Malmö, Sweden; ^3^Centre for Primary Health Care Research, Department of Clinical Sciences, Malmö, Lund University/Region Skåne, Sweden

## Abstract

**Background:**

Individuals with opioid dependence are at increased risk of deteriorating health due to the lifestyle connected to heroin use. Barriers surrounding the healthcare system seem to hinder patients to seek help through conventional healthcare, even after entering opioid substitution treatment (OST), resulting in a high level of unmet healthcare needs. However, this field is still unexplored, with only a few studies focusing on general health within this population. The first step, in order to provide suitable and accessible primary healthcare, is to assess the extent of physical symptoms and unmet healthcare needs within the OST population, which, to this point, has been sparsely studied.

**Aim:**

To assess OST patients' self-rated physical health and healthcare seeking behaviour.

**Methods:**

Two-hundred and eighteen patients from four different OST sites answered a questionnaire regarding physical health and healthcare seeking.

**Results:**

Patients in OST have a high degree of physical symptoms and a high degree of unmet healthcare needs. Sixty-six percent reported suffering from musculoskeletal pain. Fifty-six percent reported gastrointestinal symptoms. Genital problems and airway symptoms were reported by 47%, respectively, and dental problems were reported by 69% of the respondents. General unmet healthcare needs were reported by 82%. Musculoskeletal pain was positively correlated with having an unstable housing situation (AOR 4.26 [95% CI 1.73-10.48]), negatively correlated with male sex (AOR 0.45 [95% CI 0.22-0.91]), and positively correlated with age (AOR 1.04 [95% CI 1.01-1.07]). No statistically significant correlates of respiratory, gastrointestinal, genital, or dental symptoms were found.

**Conclusion:**

Patients in OST carry a heavy burden of physical symptoms and unmet healthcare needs, potentially due to societal barriers. Patients' frequent visits to the OST clinics offer a unique opportunity to build a base for easily accessible on-site primary healthcare.

## 1. Introduction

Patients in opioid substitution treatment (OST) are suspected to suffer from poor health due to prior, or ongoing, substance use [1]. Entering OST decreases morbidity and mortality [2–6]; however, years of substance use have a wide effect on the somatic health.

A diverse range of health conditions can be linked to direct consequences of the drug use itself, and to lifestyle factors associated with drug use such as homelessness, mental health issues, and violence. There is also increased morbidity as a result of nonfatal overdoses, as this can lead to both acute and chronic health conditions [7–10]. A vast majority of patients in OST have suffered from at least one well-defined nonfatal overdose [11, 12]. Additionally, preventive or primary healthcare is underutilized, due to the barriers surrounding the healthcare system, and the fact that healthcare seeking might have lower priority when there is a daily struggle finding shelter, food, and money to support substance dependence [13]. Indolent or nonurgent health issues are often postponed until they become severe [14] and may finally be addressed in emergency departments rather than in primary care [15].

Islam et al. conclude that only a few studies have examined the broader public health challenges and unmet healthcare needs within the OST population [16, 17]. Our study aims to address this gap, mapping out the base on which future targeted healthcare can be built as OST clinics provide a unique opportunity to identify and to provide healthcare and support on a holistic level.

## 2. Aim

The aim of this study is to assess OST patients' self-rated physical health and healthcare seeking behaviour.

## 3. Materials and Methods

### 3.1. Setting

The study was conducted at four OST clinics in Malmö, southern Sweden. Malmö has a population of more than 300,000 inhabitants. It is the third largest city in Sweden, and the largest city in the region of Skåne which has a population of 1.34 million.

The Swedish healthcare system is divided into somatic and psychiatric healthcare, with the latter including the field of substance dependence. The Malmö Addiction Centre, geographically located within the Malmö University Hospital, includes an emergency addiction unit, an in-patient detox ward for opioid dependence and a psychiatric emergency unit.

On a national level, availability of needle exchange program (NEP) differs immensely. NEPs in Skåne, available since the 1980s, are stationary units, run by the Department of Infectious Diseases in the university hospital area. The NEPs are providing patients with clean injection tools, basic medical care, and counselling. Patients are regularly tested for HIV and hepatitis B and C, and a vaccination program offers vaccination for hepatitis A and B.

The number of patients in OST has slowly increased in Sweden since 1966 when methadone was first introduced. OST is allowed only at specialised addiction treatment units. Availability has traditionally been restricted through high-threshold policies. Throughout the years the National Board of Health and Welfare has gradually made recommendations less restricted [18–20], aiming to increase access and availability. However, on a national level, availability differs greatly depending on geographical region. Policy changes in 2013 made Skåne the only region in Sweden experiencing a rapid change in OST access and availability. OST units grew from five to 18 units, provided by both private and public caregivers. All OST services are tax financed and covered by the Swedish universal health insurance. It is mandatory for the OST clinics in Skåne to provide for a holistic treatment perspective, through offering patients not only pharmacological treatment of opioid dependence, but also diagnosing and treating other psychiatric conditions and providing basic somatic healthcare such as wound care. Regular urinalysis and testing for HIV/hepatitis C are part of the services provided. OST staff include psychiatrists, nurses, counsellors/social workers, and psychologists. Most OST services are able to help patients with daily distribution of additional medication and weekly injections with long-lasting antipsychotic drugs. Since June 2018, overdose education and provision of take-home naloxone, free of charge, are offered to all patients attending OST and NEP services in Skåne.

Primary healthcare in Sweden is comprehensive and strongly subsidized. Both public and private caregivers are available. Primary healthcare, as well as secondary and tertiary healthcare, is tax funded and covered by a universal health insurance. All Swedish citizens are automatically registered at a specific primary healthcare centre (the one nearest to one's postal address); however it is possible to actively choose another one. Dental health is not covered by the universal health insurance. Self-paid fee in Sweden has a cost ceiling of 1100 SEK (~110 EUR) per year for healthcare appointments, and 2200 SEK (~220 EUR) for prescribed medications.

### 3.2. Study Design

For this quantitative study of self-rated physical health, we used cross-sectional questionnaire data. The study was approved by the Regional Ethics Board, Lund (file no. 2016/1105).

The questionnaire was developed by consultation among the principal investigators, based on former study CHOP Assessment RNB 7-29-15, created by Lorvick and colleagues, studying utilization of primary healthcare among individuals with severe addiction, modified to accommodate to the Swedish healthcare system. Additional questions from the Swedish National Survey on Primary Healthcare were added to the survey [21]. Questions from a regional program covering tobacco use, alcohol use, physical exercise, and nutrition habits were translated by authors (K.T. and D.D.) and added.

### 3.3. Sample

Patients in OST were randomly selected by OST staff or by one of the authors (K.T.), with the intention of offering every patient the possibility to participate in the study. Prior to patient inclusion one of the authors was visiting all four units on hours open for patients to “drop-in,” trying to target the busiest hours during the week, and answering questions from both patients and staff. To ensure that enough questionnaires were collected from the smallest unit, which had a rather large proportion of patients being in treatment institutions and hospitals, a letter of information, completed with the questionnaire, was sent to potential study participants with a stamped and addressed envelope. Exclusion criteria were psychiatric conditions or drug influence preventing the patient from giving informed consent. The study was conducted between 4 May 2017 and 6 March 2018. No economic compensation was provided for study participation. Respondents answered the questionnaire after receiving oral and written information about the study, and providing a written informed consent.

### 3.4. Data Collection

After informed consent was obtained, study participants were asked to fill out the questionnaire when visiting their OST clinic. The questionnaire included 34 mainly closed-ended questions, with the possibility to describe symptoms and contact with healthcare services. In 12 out of the 34 questions there were subsequent questions, stating “If answering ‘yes' to the previous question” one was directed to a subsequent question (A), and in 8 of these questions there was also a question “B” and “C”. The questionnaire covered four areas where section one consisted of background information regarding demographic variables, section two assessed habits concerning tobacco, alcohol, nutrition, and physical exercise, section three included questions about self-rated health and quality of life, blood-borne infections, and symptoms from the airways, mouth and teeth, gastrointestinal system, genitals, and musculoskeletal pain, and section four contained questions about healthcare seeking, health literacy, and experience from encounters with the healthcare system.

If the participants had problems understanding questions or if they found questions not covering their situation, staff at the OST clinic clarified the question. In a few cases where study participants had severe reading difficulties, they received extensive help reading and writing, as we did not exclude participants due to these circumstances.

In seven cases participants had answered the questionnaire twice. Only results from the first questionnaire were included in the results.

### 3.5. Data Analysis and Statistical Methods

Two variables were recoded. Housing situation was recoded to “unstable housing” if the respondent replied (multiple choice question) “transitional apartment,” “institution/family care placement,” “hotel,” “homeless,” or “other”. Experience of poor treatment due to OST/drug use was recoded to “yes” if the answer was “yes, sometimes” or “yes, often or always.”

When data were missing for yes/no questions regarding specific symptoms, the answer was recoded to “yes” if the participant had described symptoms in the open-ended question. Questions regarding healthcare seeking for specific symptoms were recoded as “yes” and “no,” respectively, if answer to the yes/no question was missing but the participant had described where they sought healthcare, or described reasons for not seeking healthcare.

Symptom-specific unmet healthcare need was defined as reporting symptoms but not seeking healthcare for that symptom.

General unmet healthcare needs were a computed variable, sorting out those who had unmet healthcare needs for any symptom from airways, gastrointestinal system, genitals, or musculoskeletal system. Dental symptoms were not included in this variable since these symptoms are not assessed by primary healthcare, but within the dentistry system which is not fully covered by the Swedish insurance system.

The results from the questionnaires were registered and analysed using IBM SPSS Statistics, version 24. Missing data were not recoded as “no” but were excluded from analyses. Correlates of physical symptoms were calculated by multivariate logistic regression analysis in relation to three variables: age, sex, and unstable housing. Each physical symptom was analysed as dependent variable. P-value <0.05 was considered statistically significant.

## 4. Results

### 4.1. Sample Demographics

Two hundred and eighteen patients in OST (28% female, median age 43 [range 23-67, SD 10.2 years]) were included in the study (Table 1). Twenty percent had an unstable housing situation. A majority (54%) were receiving social welfare and 17% received their main income through employment. Daily tobacco smoking was reported by 75%.

### 4.2. Self-Rated Physical Health

Self-reported current physical symptoms were common; almost every second patient reported symptoms from the airways or genital area (47%), respectively. Approximately half of the population reported gastrointestinal symptoms (56%). Sixty-six percent reported pain from arms, legs, neck, or back, and 69% dental problems (Table 2).

### 4.3. Unmet Healthcare Needs and Knowledge about Healthcare Seeking

A majority of the study population had not sought healthcare for their symptoms. Unmet healthcare needs (i.e., reporting physical symptoms but having refrained from seeking healthcare) were most common for genital symptoms (80%), followed by pain (71%) and gastrointestinal symptoms (66%) (Table 3). Unmet healthcare needs regarding respiratory and dental symptoms were 56% and 52%, respectively. General unmet healthcare needs were 82% in the population, and experience of poor treatment due to OST or drug use was reported in 64%.

Self-reported knowledge about when and how to contact primary healthcare was generally good. Eighty percent reported being aware of which primary healthcare centre they were listed at, 84% knew when to contact primary healthcare rather than emergency care or other healthcare providers, and 93% reported that they knew how to make an appointment at the primary healthcare centre.

### 4.4. Correlates of Physical Symptoms

There was no statistically significant correlation between any of the covariates sex, age, and unstable housing. Musculoskeletal pain was positively correlated with unstable housing (AOR 4.26 [95% CI 1.73-10.48]) and age (AOR 1.04 [95% CI 1.01-1.07]), and negatively correlated with male sex (AOR 0.45 [95% CI 0.22-0.91]) (Table 4). We found no statistically significant correlates of respiratory, gastrointestinal, genital, or dental symptoms.

## 5. Discussion

Access to healthcare and availability hereof are imperative to prevent diseases and maintain health in the general population. Our results show high prevalence of self-reported physical symptoms as well as a high degree of unmet healthcare needs among patients in OST. To our knowledge, physical healthcare problems within this population have only been sparsely described in international studies, with the exception of healthcare focusing on direct consequences of intravenous drug use [16, 17]. Compared to research on the general population, these numbers show a disproportionately high level of physical illness within the OST population. The high numbers of general unmet healthcare (82%) are also worrying as they indicate that a large proportion of the study participants are being subject to prolonged or unnecessary suffering.

Almost half of the study subjects reported having problems with their airways, and, of those, more than half refrained from seeking healthcare. Consistent with international findings, significantly higher prevalence of respiratory symptoms is found within the OST population [1]. Research in an Australian OST population found 30% of their sample to meet the criteria for COPD (FEV1/FVC <0.70) [22]. As the worldwide leading cause of pulmonary disease is tobacco smoking [23] one could expect to find a higher prevalence than in the general population. A vast majority (75%) of individuals included in our study were daily smokers, compared to an average of 9% in the general adult Swedish population [24]. International research on substance dependence shows tobacco smoking prevalence in 73-97%, with OST patients generally being at the higher end [25–33]. Apart from tobacco smoking, inhalation, smoking, or intravenous use of substances can cause a wide spectrum of diseases affecting the lungs, either direct or indirect. Injecting crushed oral tablets may lead to foreign-body granulomatosis as the excipient (filler material) can induce a potentially fatal, foreign body reaction in pulmonary arterioles [34–36]. Furthermore, up to 74% of heroin users in the present region have experienced at least one overdose [11, 12] which can lead to complications such as noncardiogenic pulmonary oedema and aspiration pneumonitis [36].

The prevalence of musculoskeletal pain was high (66%) in our study, which is coherent with previous research. International studies on patients in methadone maintenance treatment show a 37% prevalence of severe chronic pain [37, 38], and an 80% prevalence of nonspecified pain during the past week [37]. A systematic review on chronic pain prevalence in the general worldwide population addresses the difficulties in comparing results as chronic pain definition criteria are highly inconsistent. Included results range from 9% to 64% with a total pooled mean of 31% [39], whereas a large scale survey on chronic pain in Europe showed a 19% prevalence of moderate to severe chronic pain [40]. Chronic pain is associated with personality disorders, depression, and anxiety and somatoform disorders in the general population [41]. This is also seen in the OST population, with a high degree of psychiatric comorbidities [42, 43]. Lack of evidence-based treatments and guidelines on opioid use disorders and concurrent pain makes management more complex [44–47], which is likely to cause unmet healthcare needs [42, 44, 45, 48] and unsatisfied patients [42, 45]. This may shed a light on why a vast majority of our study subjects (71%) refrained from seeking healthcare for musculoskeletal pain. Our results on pain correlating to being older, having an unstable housing situation, and being female are in line with previous studies showing that prevalence of chronic pain is increasing with age and is more prevalent among women [49]. Homelessness has negative effects on various levels including pain, which is overrepresented among homeless people compared to the general population [50].

Gastrointestinal problems were reported by 56% of our responders, many of whom described symptoms of opioid-induced bowel dysfunction. This is a distressing condition including symptoms of nausea, vomiting, bloating, abdominal pain, gastro-oesophageal reflux-related symptoms, and constipation, among others [51]. Opioid-induced constipation is common among individuals on long-term treatment with opioids, with a prevalence among OST patients exceeding 60% [52]. The mean value of reported constipation rates in the general European population was 17% [53] and self-reported prevalence of constipation in a general Swedish population was 20% among women and 8% among men [54]. Desire in minimizing additional medical intake and not being satisfied with the result of constipation medication has shown to be common reasons for not using medication, in a general population [55]. However, it hardly explains why a majority (66%) of the respondents, in the current study, had refrained from seeking treatment for their gastrointestinal symptoms.

Health issues within the area of gynaecological or sexual dysfunction were reported by 47% of our study participants. An international systematic review shows that the proportion of sexual dysfunction in the general female population varies from 26% to 91% [56]. Petherick concludes that research on women's sexual desire, satisfaction, and dysfunction is still scarce, compared to men's [57]. A meta-analysis of sexual dysfunction among male patients on methadone treatment displayed a pooled prevalence of 52%, ranging from 16% to 84% [58]. In the present study, the high frequency of missing response on questions within this area gives us reason to believe that our result is not covering the full scope of the problem. It is troublesome that as many as 80% of our respondents reported that they had not sought healthcare for their problems as studies on sexual dysfunction have shown an increased risk of OST drop-out or an increased use of drugs with the aim of enhancing the sexual ability [59]. Sexual dysfunction may also affect relationships and lead to alienation and loneliness, which can also have a great impact on quality of life [59].

A large number of individuals in the current study reported dental problems (69%), which is consistent with international reviews showing a high prevalence of oral symptoms, among drug using individuals, which are caused by multiple factors [60, 61]. One central factor is attributed to the analgesic effects of opioids which can mask pain associated with caries and oral infection, leading to a delay in dental care seeking resulting in need of more invasive treatments once dental care is sought [60–62]. Research by Charnock et al. showed that 68% of clients visiting the Community Drug and Alcohol team reported currently having problems concerning their oral health. While 29% reported consider themselves as regular attenders, 46% reported only visiting the dentist when in pain. Main reasons for not visiting the dentist when needed were described as “fear of the dentist” (57%), “dentist being unsympathetic” (50%), “not affording dental care” (32%), whereas 28% where not sure about the reason why access was problematic [63]. International studies point to financial barriers as being the main reason behind a large and growing unmet oral healthcare need in the United States [64, 65]. Self-reported data on health and oral health in a general Swedish population showed that the lower the socioeconomic position, the higher the risk of experiencing poor oral health and poor general health [66]. Dental care is not covered by the Swedish health insurance but can be subsidized with a social benefit for certain interventions and up to a certain limit of expenditure. This is reflected in our study as a majority of the 52% reported that they had not sought treatment because they could not afford to, but in many cases the reasons might be more complex. However, the study by Charnock and colleagues showed that access to dental care can be improved, offering a one-session/week by a dentist and an oral health promotion officer on-site [63].

Unmet healthcare needs of low-income households in Europe are five times higher than those of high-income households [67]. In Sweden, these numbers are three to one [67] and even though public health seems to be improving generally in Sweden, health inequity increases [68]. Individual socioeconomic position plays a major part when it comes to risk of illness and degree of access to prevention and treatment [68]. Low socioeconomic position is one aspect on why individuals included in our study had a high degree of general unmet healthcare needs (82%). Nearly two thirds of our study participants reported experiencing worse care, because of drug use or being in OST. Stigma and structural barriers within the healthcare system seem to cover further aspects of the problem. These barriers have been described in previous research in the realm of other priorities; fear of disease or stigma and ignorance are interwoven on different levels of society and are known to be hindering marginalized patients from seeking healthcare [13, 16].

Public stigma has been described as a result of social construction of addiction, in which self-stigma is a result of internalization of constructed norms, leading to interpretation of oneself as a failure [69]. Stigma may limit patients from utilizing healthcare, but it may also limit caregivers from giving patients equal care, considering the individual's needs. Research on stigma, in relation to addiction, and its negative consequences on health and quality of life, is sparse [70]. However, research shows that being a methadone patient was perceived to be more stigmatizing than injecting opioids, creating barriers both towards OST and to patients in treatment [71]. Stigma and fear of sanctions from society and the legal apparatus [72, 73] may hinder or delay patients from the utilization of the healthcare system. Medical staff's limited knowledge of and confidence regarding healthcare for substance dependence [74], combined with unfavourable attitudes [75], fear of deception and inconsistency [76] or that patients will be difficult, aggressive, or demanding [74], fuels patients' experience of being stigmatized and not receiving proper treatment [76]. However, education on substance use and dependence has been shown to lead to changes in attitudes of medical staff, and subsequently reduction of stigma [75, 77].

Generally, healthcare systems operate in separate systems of care [78] relying on patients being compliant, and having a sufficient level of health literacy and self-sufficiency. These demands may lead to an increased degree of unmet healthcare needs in marginalized populations. Studies show that individuals with substance use often misunderstand their medical condition [79] and have difficulties in keeping appointments within the general healthcare system [80]. Congruent with results from our study it seems clear that there is a need to find ways to override the barriers perceived by a large proportion of OST patients. Our results have important clinical implications.

Provision of on-site primary healthcare has been shown to improve OST patients' access to healthcare as diversified health services are offered in a user-friendly environment. A randomized controlled trial study conducted at an OST clinic showed that nearly all patients (92%) received healthcare on-site, compared to 35% of patients who were referred to other healthcare facilities [79]. OST patients' regular contact with their clinic provides a unique opportunity to provide both planned and acute primary healthcare visits on-site [79, 81]. The extent of provision of on-site primary healthcare is significantly associated with greater utilization of primary healthcare, reducing emergency department visits and inpatient treatment [81, 82]. A high degree of access to primary healthcare is associated with improved health outcomes and cost-effectiveness [83, 84], especially when it comes to disadvantaged populations [85]. The flexibility within on-site primary healthcare has the ability to override barriers when it comes to difficulties connected to availability and access [16]. However it also offers an opportunity for patients to obtain healthcare without fear of stigma. Providing healthcare on-site in a familiar environment, without judgement or fear of sanctions, may increase engagement with services [79, 81].

## 6. Strengths and Limitations

The study design, being observational and cross-sectional, allows analysis of associations, but not of cause and effect. Using self-reported data does have certain limitations due to response or recall bias, potentially leading to under- or overestimations. However, this method is thought to be the best method when it comes to studying patients' experiences and perceived needs [86, 87]. Reliability in open-ended questions appeared to be low, which made analyses hereof impossible. Representability of the studied population seems to be acceptably high as 46% of all patients enrolled in one of the four OST sites were included during the study period. Age and gender among the study subject were similar to all participants enrolled in the four OST clinics during the study period. The slight difference in median age between subjects included in the study and the whole OST population, 43 and 45 years, respectively, could indicate that included individuals to a larger extent are represented by patients visiting the units more frequently, while older patients tend to visit the units more rarely. However, representation of women among individuals included and of the total population was 28%, respectively. Regional differences may, however, provide differences in populations, which also leads to lower generalizability.

## 7. Conclusions

Patients in OST have a high degree of self-reported physical symptoms and a high degree of unmet healthcare needs. On-site primary healthcare has potential to reduce healthcare seeking barriers and improve OST patients' physical health. Further research evaluating on-site primary healthcare is needed, as is exploring reasons behind the large proportion of unmet healthcare needs as well as attitudes among medical staff.

## Figures and Tables

**Table 1:** Patient characteristics, N=218. *(Missing values excluded from the denominator).*

Characteristics	All participants	OST Bokgatan	OST Hasselgatan	OST Matris	OST INM
Total n	218	47	81	42	48
Age mean	43.9	48.1	44.4	40.6	41.8
(range; S.D.)	(23-67; 10.2)	(27-67; 10.7)	(23-62; 8.8)	(25-57; 9.8)	(24-63; 10.9)
Age median	43	47	44	38.5	41
Female sex	61 (28%)	12 (26%)	27 (33%)	9 (21%)	13 (27%)
Born in Sweden	165 (76%)^2^	35 (76%)^1^	60 (74%)	32 (76%)	38 (81%)^1^
Unstable housing	43 (20%)^4^	4 (9%)	16 (20%)^2^	14 (33%)	9 (20%)^2^
Main source of income					
Social services	114 (54%)	25 (54%)	38 (48%)	24 (62%)	27 (58%)
Sick leave	15 (7%)	1 (2%)	5 (6%)	4 (10%)	5 (11%)
Permanent sick leave	31 (15%)	8 (17%)	13 (16%)	4 (10%)	6 (13%)
Employment	35 (17%)	6 (13%)	18 (23%)	5 (13%)	6 (13%)
Retirement	4 (2%)	4 (9%)	0	0	0
Other	13 (6%)	2 (4%)	6 (8%)	2 (5%)	3 (6%)
missing	6	1	1	3	1
Daily tobacco smoking	156 (75%)^5^	35 (78%)^2^	59 (73%)	30 (77%)^3^	32 (73%)^4^

^1^missing n=1 ^2^missing n=2 ^3^missing n=3 ^4^missing n=4 ^5^missing n=9.

**Table 2:** Self-reported physical symptoms, N=218.

Health characteristic	Valid n	n (% of valid)
Respiratory symptoms	204	96 (47%)
Pain from extremities, back or neck	210	138 (66%)
Gastrointestinal symptoms	205	115 (56%)
Genital symptoms	207	98 (47%)
Dental symptoms	206	142 (69%)

*(Missing values excluded from the denominator).*

**Table 3:** *Unmet healthcare needs*. Total n=218. Percentage of valid answers. Excluded - no symptoms or missing answer.

Health characteristic	Valid n	n (% of valid)
General unmet healthcare needs		
(respiratory, pain, gastrointestinal, genital)	188	154 (82%)
Did not seek healthcare for respiratory symptoms	89	50 (56%)
Did not seek healthcare for pain	130	92 (71%)
Did not seek healthcare for gastrointestinal symptoms	100	66 (66%)
Did not seek healthcare for genital symptoms	93	74 (80%)
Did not seek healthcare for dental symptoms	128	66 (52%)

Worries about physical health, did not seek healthcare	103	44 (43%)
Healthcare need but refrained from seeking, past year.	198	105 (53%)

Aware of which primary care listed to	202	162 (80%)
Aware of when to seek primary care	203	170 (84%)
Aware how to contact primary care	203	188 (93%)
Experience of poor treatment due to OST/drug use	206	131 (64%)

**Table 4:** Correlates of physical symptoms. Multivariable logistic regression. Adjusted odds ratio (AOR [95% CI]).

Covariate	Airways		Pain		Gastrointestinal		Genital		Dental	
	(n=201)		(n=208)		(n=202)		(n=205)		(n=204)	
	p	AOR	p	AOR	p	AOR	p	AOR	p	AOR
Age	0.09	1.03 (1.00-1.05)	**0.02** *∗*	1.04 (1.01-1.07)	0.67	1.01 (0.98-1.04)	0.85	1.00 (0.98-1.03)	0.08	1.03 (1.00-1.06)
Male sex	0.38	0.75 (0.40-1.42)	**0.03** *∗*	0.45 (0.22-0.91)	0.11	0.59 (0.31-1.13)	0.72	0.89 (0.48-1.65)	0.21	0.64 (0.32-1.29)
Unstable housing	0.39	1.37 (0.67-2.80)	**0.002** *∗∗*	4.26 (1.73-10.48)	0.74	0.99 (0.96-1.03)	0.72	0.88 (0.44-1.76)	0.38	1.41 (0.65-3.04)

CI= confidence interval *∗* p<0.05  *∗∗* p<0.01.

## Data Availability

The SPSS data used to support the findings of this study are restricted by the Regional Ethics Board, Lund, Sweden, in order to protect patient privacy. Data are available from Katja Troberg, katja.troberg@med.lu.se, for researchers who meet the criteria for access to confidential data.

## References

[1] O’Toole J., Hambly R., Cox A., O’Shea B., Darker C. (2014). Methadone-maintained patients in primary care have higher rates of chronic disease and multimorbidity, and use health services more intensively than matched controls. *European Journal of General Practice*.

[2] Gronbladh L., Ohlund L. S., Gunne L. M. (1990). Mortality in heroin addiction: impact of methadone treatment. *Acta Psychiatrica Scandinavica*.

[3] Caplehorn J. R., Dalton M. Y., Haldar F., Petrenas A., Nisbet J. G. (2009). Methadone maintenance and addicts' risk of fatal heroin overdose. *Substance Use & Misuse*.

[4] Brugal M. T., Domingo-Salvany A., Puig R., Barrio G., de García Olalla P., de La Fuente L. (2005). Evaluating the impact of methadone maintenance programmes on mortality due to overdose and aids in a cohort of heroin users in Spain. *Addiction*.

[5] Kakko J., Grönbladh L., Svanborg K. D. (2007). A stepped care strategy using buprenorphine and methadone versus conventional methadone maintenance in heroin dependence: a randomized controlled trial. *The American Journal of Psychiatry*.

[6] Mattick R. P., Breen C., Kimber J., Davoli M. (2009). Methadone maintenance therapy versus no opioid replacement therapy for opioid dependence. *Cochrane Database of Systematic Reviews*.

[7] Darke S., Sims J., McDonald S., Wickes W. (2000). Cognitive impairment among methadone maintenance patients. *Addiction*.

[8] Sporer K. A., Firestone J., Isaacs S. M. (1996). Out-of-hospital treatment of opioid overdoses in an urban setting. *Academic Emergency Medicine*.

[9] Boyer E. W. (2012). Management of opioid analgesic overdose. *The New England Journal of Medicine*.

[10] Warner-Smith M., Darke S., Day C. (2002). Morbidity associated with non-fatal heroin overdose. *Addiction*.

[11] Bråbäck M., Nilsson S., Isendahl P., Troberg K., Brådvik L., Håkansson A. (2016). Malmö Treatment Referral and Intervention Study (MATRIS)—effective referral from syringe exchange to treatment for heroin dependence: a pilot randomized controlled trial. *Addiction*.

[12] Brådvik L., Hulenvik P., Frank A., Medvedeo A., Berglund M. (2007). Self‐reported and observed heroin overdoses in Malmoe. *Journal of Substance Use*.

[13] Carr S., Goldberg D. J., Elliott L., Green S., Mackie C., Gruer L. (2010). A primary health care service for Glasgow street sex workers-6 years experience of the 'Drop-in Centre', 1989-1994. *AIDS Care*.

[14] McCoy C. B., Metsch L. R., Chitwood D. D., Miles C. (2001). Drug use and barriers to use of health care services. *Substance Use & Misuse*.

[15] Laine C., Lin Y.-T., Hauck W. W., Turner B. J. (2005). Availability of medical care services in drug treatment clinics associated with lower repeated emergency department use. *Medical Care*.

[16] Islam M. M., Topp L., Day C. A., Dawson A., Conigrave K. M. (2012). The accessibility, acceptability, health impact and cost implications of primary healthcare outlets that target injecting drug users: A narrative synthesis of literature. *International Journal of Drug Policy*.

[17] Mofizul Islam M., Topp L., Day C. A., Dawson A., Conigrave K. M. (2012). Primary healthcare outlets that target injecting drug users: Opportunity to make services accessible and acceptable to the target group. *International Journal of Drug Policy*.

[18] The National Board of Health and Welfare (Socialstyrelsen). Socialstyrelsens föreskrifter och allmänna råd om läkemedelsassisterad behandling vid opioidberoende (The Board's provisions and general guidelines on medication-assisted treatment in opioid dependence), HSLF-FS, vol. 2016, p. 1, 2016

[19] Fugelstad A., Stenbacka M., Leifman A., Nylander M., Thiblin I. (2007). Methadone maintenance treatment: the balance between life-saving treatment and fatal poisonings. *Addiction*.

[20] Romelsjö A., Engdahl B., Stenbacka M. (2010). Were the changes to Sweden's maintenance treatment policy 2000-06 related to changes in opiate-related mortality and morbidity?. *Addiction*.

[21] National patient survey on Primary Healthcare https://patientenkat.se/sv/resultat/primarvard-2017/.

[22] Islam M. M., Taylor A., Smyth C., Day C. A. (2013). General health of opioid substitution therapy clients. *Internal Medicine Journal*.

[23] WHO Tobacco http://www.who.int/news-room/fact-sheets/detail/tobacco.

[24] The Public Health Agency of Sweden (Folkhälsomyndigheten). Tobak (Tobacco), Available at: http://www.folkhalsomyndigheten.se/folkhalsorapportering-statistik/folkhalsans-utveckling/levnadsvanor/tobaksrokning-daglig/, 2018

[25] Zirakzadeh A., Shuman C., Stauter E., Hays J. T., Ebbert J. O. (2013). Cigarette smoking in methadone maintained patients: an up-to-date review. *Current Drug Abuse Reviews*.

[26] Okoli C. T., Khara M., Procyshyn R. M., Johnson J. L., Barr A. M., Greaves L. (2010). Smoking cessation interventions among individuals in methadone maintenance: A brief review. *Journal of Substance Abuse Treatment*.

[27] Nahvi S., Richter K., Li X., Modali L., Arnsten J. (2006). Cigarette smoking and interest in quitting in methadone maintenance patients. *Addictive Behaviors*.

[28] Richter K. P., Gibson C. A., Ahluwalia J. S., Schmelzle K. H. (2001). Tobacco use and quit attempts among methadone maintenance clients. *American Journal of Public Health*.

[29] Guydish J., Passalacqua E., Pagano A. (2016). An international systematic review of smoking prevalence in addiction treatment. *Addiction*.

[30] Apollonio D., Philipps R., Bero L. Interventions for tobacco use cessation in people in treatment for or recovery from substance use disorders. *Cochrane Database of Systematic Reviews*.

[31] Mendelsohn C. P., Wodak Am A. (2016). Smoking cessation in people with alcohol and other drug problems. *Australian Family Physician*.

[32] Pajusco B., Chiamulera C., Quaglio G. (2012). Tobacco addiction and smoking status in heroin addicts under methadone vs. buprenorphine therapy. *International Journal of Environmental Research and Public Health*.

[33] Shah P. A., Cunningham C. O., Brisbane M. T., DeLuca J. P., Nahvi S. (2017). Use of smoking cessation methods among patients receiving office-based buprenorphine maintenance treatment. *Journal of Addiction Medicine*.

[34] Nguyen V. T., Chan E. S., Chou S. S. (2014). Pulmonary effects of iv injection of crushed oral tablets: “excipient lung disease”. *American Journal of Roentgenology*.

[35] Bishay A., Amchentsev A., Saleh A., Patel N., Travis W., Raoof S. (2008). A hitherto unreported pulmonary complication in an iv heroin user. *CHEST*.

[36] Mégarbane B., Chevillard L. (2013). The large spectrum of pulmonary complications following illicit drug use: Features and mechanisms. *Chemico-Biological Interactions*.

[37] Rosenblum A. (2003). Prevalence and characteristics of chronic pain among chemically dependent patients in methadone maintenance and residential treatment facilities. *Journal of the American Medical Association*.

[38] Barry D. T., Beitel M., Garnet B., Joshi D., Rosenblum A., Schottenfeld R. S. (2009). Relations among psychopathology, substance use, and physical pain experiences in methadone-maintained patients. *Journal of Clinical Psychiatry*.

[39] Steingrímsdóttir Ó. A., Landmark T., Macfarlane G. J., Nielsen C. S. (2017). Defining chronic pain in epidemiological studies. *PAIN*.

[40] Breivik H., Collett B., Ventafridda V., Cohen R., Gallacher D. (2006). Survey of chronic pain in Europe: prevalence, impact on daily life, and treatment. *European Journal of Pain*.

[41] Dersh J., Polatin P. B., Gatchel R. J. (2002). Chronic pain and psychopathology: research findings and theoretical considerations. *Psychosomatic Medicine*.

[42] Jamison R. N., Kauffman J., Katz N. P. (2000). Characteristics of methadone maintenance patients with chronic pain. *Journal of Pain and Symptom Management*.

[43] Dhingra L., Masson C., Perlman D. C. (2013). Epidemiology of pain among outpatients in methadone maintenance treatment programs. *Drug and Alcohol Dependence*.

[44] Delorme J., Chenaf C., Bertin C. (2018). Chronic pain opioid-maintained patients receive less analgesic opioid prescriptions. *Frontiers in Psychiatry*.

[45] Dunn K. E., Brooner R. K., Clark M. R. (2014). Severity and interference of chronic pain in methadone-maintained outpatients. *Pain Medicine*.

[46] Dennis B. B., Bawor M., Paul J. (2016). Pain and opioid addiction: A systematic review and evaluation of pain measurement in patients with opioid dependence on methadone maintenance Treatment. *Current drug abuse reviews*.

[47] Berg K. M., Arnsten J. H., Sacajiu G., Karasz A. (2009). Providers’ experiences treating chronic pain among opioid-dependent drug users. *Journal of General Internal Medicine*.

[48] Nordmann S., Vilotitch A., Lions C. (2017). Pain in methadone patients: Time to address undertreatment and suicide risk (ANRS-Methaville trial). *PLoS ONE*.

[49] Tsang A., Von Korff M., Lee S. (2008). Common chronic pain conditions in developed and developing countries: gender and age differences and comorbidity with depression-anxiety disorders. *The Journal of Pain*.

[50] Fisher R., Ewing J., Garrett A., Harrison E. K., Lwin K. K., Wheeler D. W. (2013). The nature and prevalence of chronic pain in homeless persons: an observational study. *F1000Research*.

[51] Brock C., Olesen S. S., Olesen A. E., Frøkjaer J. B., Andresen T., Drewes A. M. (2012). Opioid-induced bowel dysfunction. *Drugs*.

[52] Haber P. S., Elsayed M., Espinoza D., Lintzeris N., Veillard A., Hallinan R. (2017). Constipation and other common symptoms reported by women and men in methadone and buprenorphine maintenance treatment. *Drug and Alcohol Dependence*.

[53] Peppas G., Alexiou V. G., Mourtzoukou E., Falagas M. E. (2008). Epidemiology of constipation in Europe and Oceania: a systematic review. *BMC Gastroenterology*.

[54] Walter S., Hallbook O., Gotthard R., Bergmark M., Sjodahl R. (2002). A population-based study on bowel habits in a Swedish community: prevalence of faecal incontinence and constipation. *Scandinavian Journal of Gastroenterology*.

[55] Ducrotté P., Milce J., Soufflet C., Fabry C. (2016). Prevalence and clinical features of opioid-induced constipation in the general population: A French study of 15,000 individuals. *United European Gastroenterology Journal*.

[56] Khajehei M., Doherty M., Tilley P. J. (2015). An update on sexual function and dysfunction in women. *Archives of Women's Mental Health*.

[57] Petherick A. (2017). Sexual arousal: sex matters. *Nature*.

[58] Yee A., Loh H. S., Hisham Hashim H. M., Ng C. G. (2014). The prevalence of sexual dysfunction among male patients on methadone and buprenorphine treatments: a meta-analysis study. *The Journal of Sexual Medicine*.

[59] Xia Y., Zhang D., Li X. (2013). Sexual dysfunction during methadone maintenance treatment and its influence on patient's life and treatment: A qualitative study in South China. *Psychology, Health & Medicine*.

[60] Brondani M., Park P. E. (2011). Methadone and oral health—a brief review. *Journal of Dental Hygiene*.

[61] Titsas A., Ferguson M. M. (2002). Impact of opioid use on dentistry. *Australian Dental Journal*.

[62] Robinson P. G., Acquah S., Gibson B. (2005). Drug users: oral health-related attitudes and behaviours. *British Dental Journal*.

[63] Charnock S., Owen S., Brookes V., Williams M. (2004). A community based programme to improve access to dental services for drug users. *British Dental Journal*.

[64] Mertz E. A. (2016). The dental–medical divide. *Health Affairs*.

[65] Vujicic M., Buchmueller T., Klein R. (2016). Dental care presents the highest level of financial barriers, compared to other types of health care services. *Health Affairs*.

[66] Hakeberg M., Wide Boman U. (2018). Self-reported oral and general health in relation to socioeconomic position. *BMC Public Health*.

[67] OECD Health at a Glance: Europe 2018 (2018). *State of Health in the EU Cycle*.

[68] The Public Health Agency of Sweden (Folkhälsomyndigheten). Public Health Report 2017, Available at: https://www.folkhalsomyndigheten.se/contentassets/9de83d1af6ce4a429e833d3c8d7ccf85/folkhalsans-utveckling-arsrapport-2017-16136-webb2.pdf, 2018

[69] Matthews S., Dwyer R., Snoek A. (2017). Stigma and self-stigma in addiction. *Journal of Bioethical Inquiry*.

[70] Corrigan P. W., Schomerus G., Shuman V. (2017). Developing a research agenda for reducing the stigma of addictions, part II: Lessons from the mental health stigma literature. *American Journal on Addictions*.

[71] Paquette C. E., Syvertsen J. L., Pollini R. A. (2018). Stigma at every turn: Health services experiences among people who inject drugs. *International Journal of Drug Policy*.

[72] Bluthenthal R. N., Kral A. H., Lorvick J., Watters J. K. (1997). Impact of law enforcement on syringe exchange programs: A look at Oakland and San Francisco. *Medical Anthropology Quarterly*.

[73] Leis R., Rosenbloom D. (2009). The road from addiction recovery to productivity: Ending discrimination against people with alcohol and drug problems. *Family Court Review*.

[74] Abouyanni G., Stevens L., Harris M. (2000). GP attitudes to managing drug- and alcohol-dependent patients: a reluctant role. *Drug and Alcohol Review*.

[75] Silins E., Conigrave K., Rakvin C., Dobbins T., Curry K. (2007). The influence of structured education and clinical experience on the attitudes of medical students towards substance misusers. *Drug and Alcohol Review*.

[76] Merrill J. O., Rhodes L. A., Deyo R. A., Marlatt G. A., Bradley K. A. (2002). Mutual mistrust in the medical care of drug users: the keys to the narc cabinet. *Journal of General Internal Medicine*.

[77] Matthews J., Kadish W., Barrett S. V., Mazor K., Field D., Jonassen J. (2002). The impact of a brief interclerkship about substance abuse on medical students' skills. *Academic Medicine: Journal of the Association of American Medical Colleges*.

[78] Samet J. H., Friedmann P., Saitz R. (2001). Benefits of linking primary medical care and substance abuse services: Patient, provider, and societal perspectives. *JAMA Internal Medicine*.

[79] Umbricht-Schneiter A., Ginn D. H., Pabst K. M., Bigelow G. E. (1994). Providing medical care to methadone clinic patients: Referral vs on-site care. *American Journal of Public Health*.

[80] Day C. A., White B., Thein H. H. (2008). Experience of hepatitis C testing among injecting drug users in Sydney, Australia. *AIDS Care*.

[81] Gourevitch M. N., Chatterji P., Deb N., Schoenbaum E. E., Turner B. J. (2007). On-site medical care in methadone maintenance: associations with health care use and expenditures. *Journal of Substance Abuse Treatment*.

[82] Friedmann P. D., Hendrickson J. C., Gerstein D. R., Zhang Z., Stein M. D. (2006). Do mechanisms that link addiction treatment patients to primary care influence subsequent utilization of emergency and hospital care?. *Medical Care*.

[83] Parthasarathy S., Mertens J., Moore C., Weisner C. (2003). Utilization and cost impact of integrating substance abuse treatment and primary care. *Medical Care*.

[84] Weisner C., Mertens J., Parthasarathy S., Moore C., Lu Y. (2001). Integrating primary medical care with addiction treatment: A randomized controlled trial. *Journal of the American Medical Association*.

[85] Starfield B., Shi L., Macinko J. (2005). Contribution of primary care to health systems and health. *Milbank Quarterly*.

[86] Bombak A. E. (2013). Self-rated health and public health: a critical perspective. *Frontiers in Public Health*.

[87] Bhandari A., Wagner T. (2006). Self-reported utilization of health care services: Improving measurement and accuracy. *Medical Care Research and Review*.

